# Supplementary material for: A Novel Isaindigotone Derivative Displays Better Anti-Proliferation Activities and Induces Apoptosis in Gastric Cancer Cells
Source: Int J Mol Sci. 2022 Jul 21;23(14):8028. doi: 10.3390/ijms23148028 (PMC9324172; doi:10.3390/ijms23148028)
Supplement: Supplementary file 1 [file ijms-23-08028-s001.zip › ijms-1790143-supplementary.pdf]

# Supplement Material

## A novel isaindigotone derivative displays better anti-proliferation activities and induces apoptosis in gastric cancer cells

Kangjia Du#, Chengjie Yang#, Zhongkun Zhou, Yunhao Ma, Yanan Tian, Rentao Zhang, Hao Zhang, Xinrong Jiang, Hongmei Zhu, Huanxiang Liu, Peng Chen *, Yingqian Liu*

School of Pharmacy, Lanzhou University, 199 Donggang West Road, Lanzhou, 730000, PR China.

The chemical atlas data of BLG26.

$^{1}$H NMR, $^{13}$C NMR, HRESIMS and HPLC Spectra of BLG26

BLG26 displays antiproliferative activity against SMMC-7721, PANC-1 and HCT116 cells.

BLG26 can get into AGS and HGC27 cells.

The yield was 69%. The m.p. was 231-233°C. Then, the NMR and ESI mass

---

# These authors contributed equally to this work.

∗ Correspondence and requests for materials should be addressed to Peng Chen (email: chenpeng@lzu.edu.cn) or Yingqian Liu (email: yqliu@lzu.edu.cn). Tel. & fax: +86 931 8915686.

spectrometry were analysed. $^{1}$H NMR (400 MHz, Chloroform-*d*) $\delta$: 7.76 (d, $J$ = 12.9 Hz, 1H), 7.61 (d, $J$ = 3.1 Hz, 1H), 7.32 - 7.24 (m, 1H), 7.23 - 7.12 (m, 2H), 7.08 (d, $J$ = 7.8 Hz, 1H), 4.20 (t, $J$ = 7.1 Hz, 2H), 3.24 (t, $J$ = 4.8 Hz, 4H), 3.17 (d, $J$ = 9.8 Hz, 2H), 2.56 (t, $J$ = 4.9 Hz, 4H), 2.31 (s, 3H). $^{13}$C NMR (100 MHz, Chloroform-*d*) $\delta$: 159.11, 154.14 (d, $J$ = 44.5 Hz), 151.88, 150.65 (d, $J$ = 12.7 Hz), 148.16 (d, $J$ = 16.1 Hz), 146.51, 145.35 (d, $J$ = 10.0 Hz), 131.67, 126.89, 125.27, 117.06, 116.89, 116.73, 114.17 (d, $J$ = 3.6 Hz), 113.51 (d, $J$ = 8.4 Hz), 111.04 (d, $J$ = 23.7 Hz), 53.89, 48.91 (d, $J$ = 4.7 Hz), 45.09, 42.93, 24.38. Purity 99.5% by HPLC. HRESIMS-ESI m/z: calcd for $C_{23}H_{21}F_3N_4O[M+H]^+$: 427.1667; found: 427.1769.

**Figure S1.** $^{1}H$ NMR spectra of BLG26.

**Figure S2.** $^{13}C$ NMR spectra of BLG26.

**Figure S3.** HRESIMS spectra of BLG26.

**Figure S4.** HPLC spectra of BLG26.

**Figure S5.** BLG26 displays anti-proliferative activity against SMMC-7721, PANC-1 and HCT116 cells. (A) BLG26 suppresses the growth of SMMC-7721 cells at 48 h. (B) BLG26 suppresses the growth of HCT116 cells at 48 h. (C) BLG26 suppresses the growth of PANC-1 cells at 48 h.

**Figure S6.** BLG26 can get into AGS and HGC27 cells. (A) Excitation spectrum of BLG26. (B) Emission spectrum of BLG26. (C) Treated AGS cells and HGC27 cells showed fluorescence.

# Figure S1.

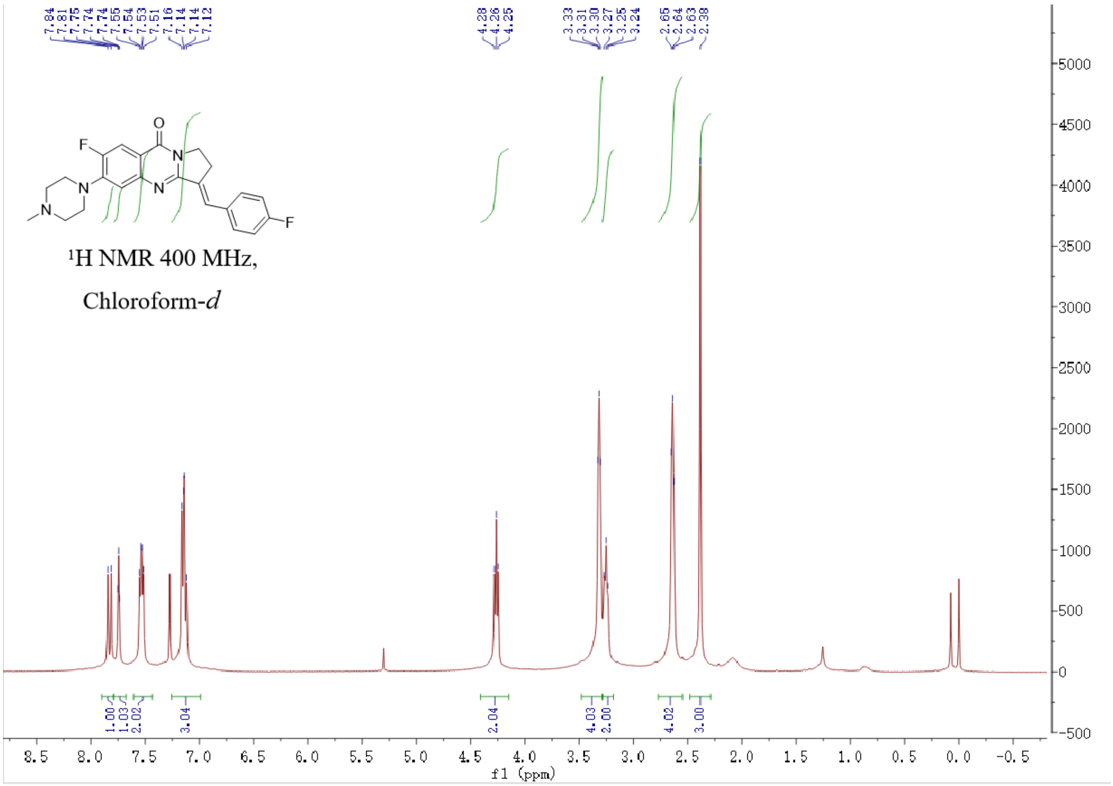

7.84
7.81
7.75
7.74
7.74
7.55
7.54
7.53
7.51
7.16
7.14
7.14
7.12
4.28
4.26
4.25
3.33
3.31
3.30
3.27
3.25
3.24
2.65
2.64
2.63
2.38
O
F
N
N
N
N
F
¹H NMR 400 MHz,
Chloroform-d
1.00
1.03
2.02
3.04
2.04
4.03
2.00
4.02
3.00
f1 (ppm)

# Figure S2.

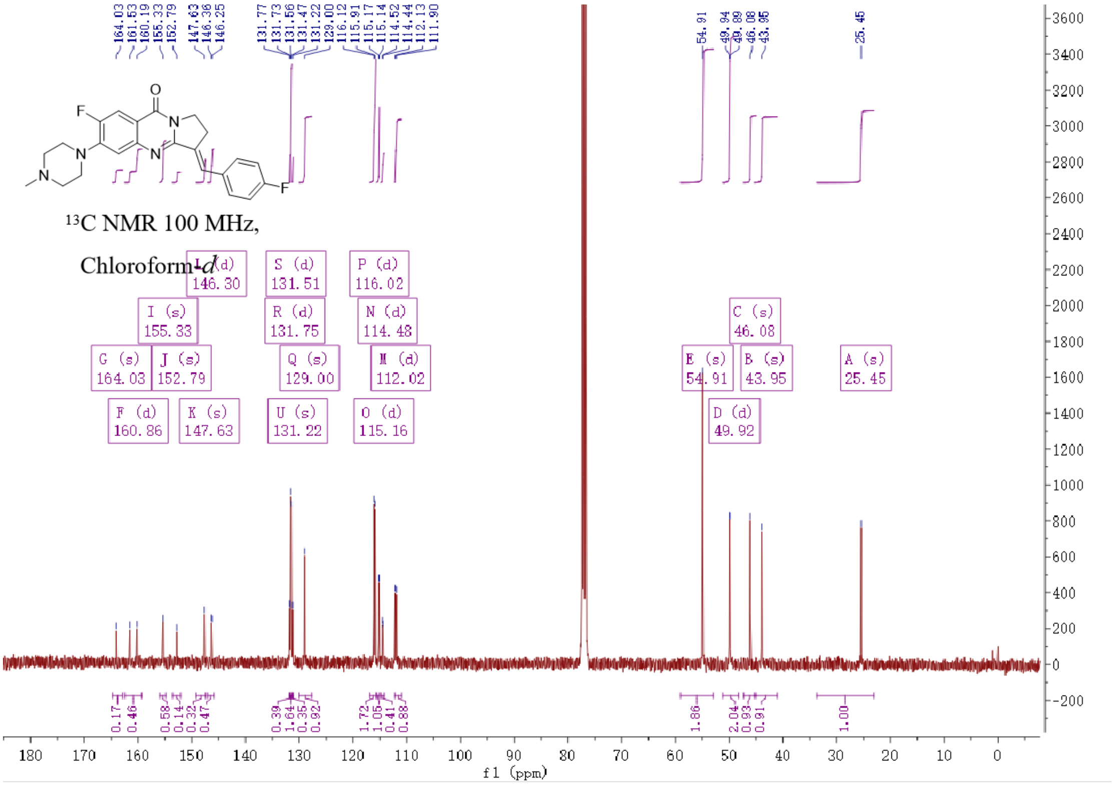

# Figure S3.

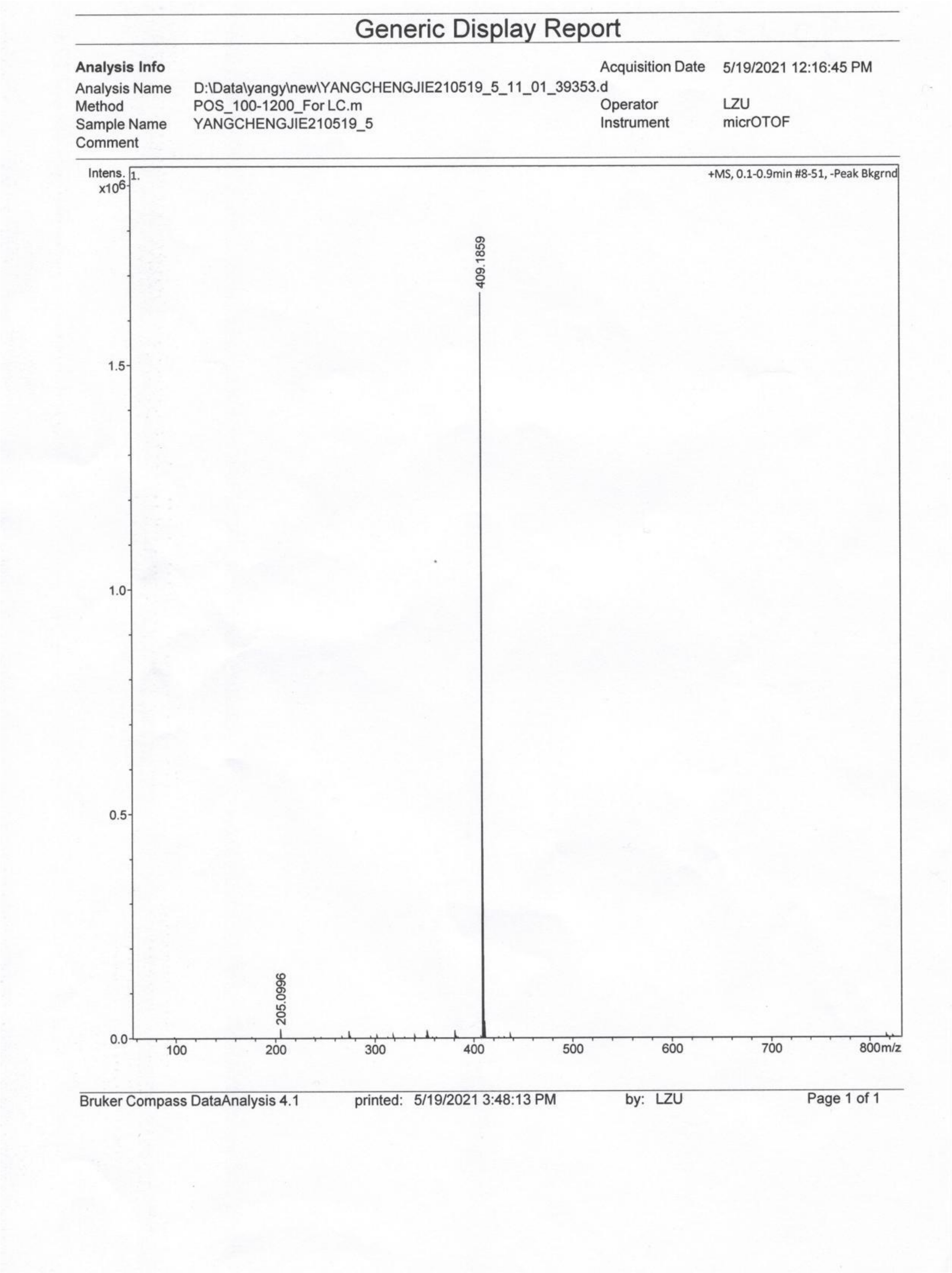
Generic Display Report
Analysis Info
Acquisition Date 5/19/2021 12:16:45 PM
Analysis Name D:\Data\yangy\new\YANGCHENGJIE210519_5_11_01_39353.d
Method POS_100-1200_For LC.m
Operator LZU
Sample Name YANGCHENGJIE210519_5
Instrument micrOTOF
Comment
Intens. x10^6
+MS, 0.1-0.9min #8-51, -Peak Bkgrnd
409.1859
205.0996
0.0
0.5
1.0
1.5
100
200
300
400
500
600
700
800m/z
Bruker Compass DataAnalysis 4.1
printed: 5/19/2021 3:48:13 PM
by: LZU
Page 1 of 1

**Figure S4.**

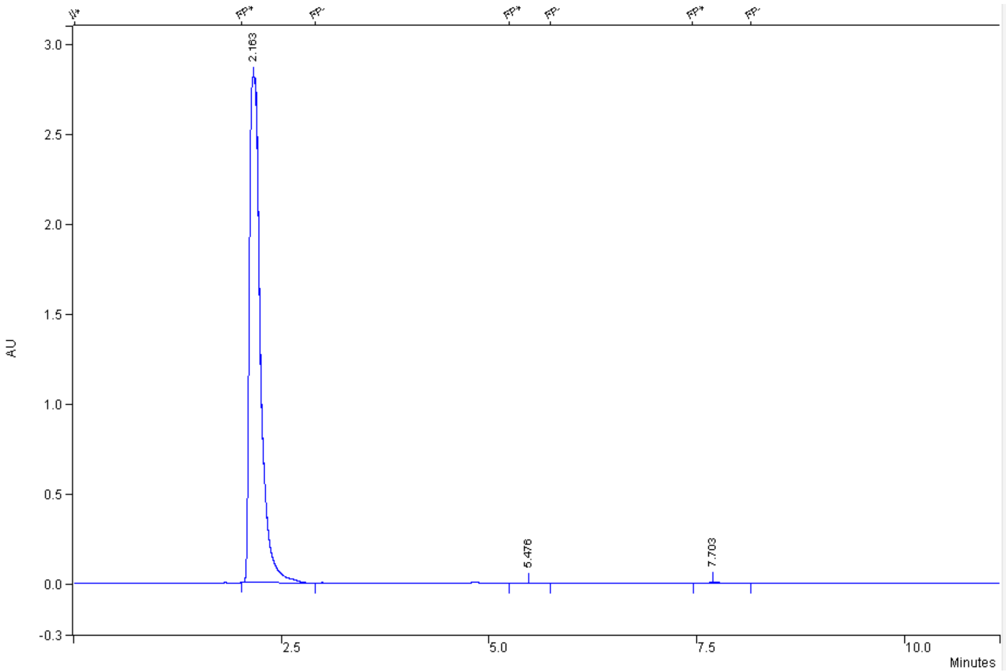


| Peak No. | Peak Name | Result () | Ret. Time (min) | Time Offset (min) | Area (counts) | Sep. Code | Width 1/2 (sec) | Status Codes |
|---|---|---|---|---|---|---|---|---|
| 1 | | 99.5622 | 2.163 | 0.000 | 260839888 | BB | 7.9 | |
| 2 | | 0.1514 | 5.476 | 0.000 | 396529 | BB | 12.2 | |
| 3 | | 0.2864 | 7.703 | 0.000 | 750338 | BB | 15.1 | |
| | Totals: | 100.0000 | | 0.000 | 261986755 | | | |

**Figure S5.**

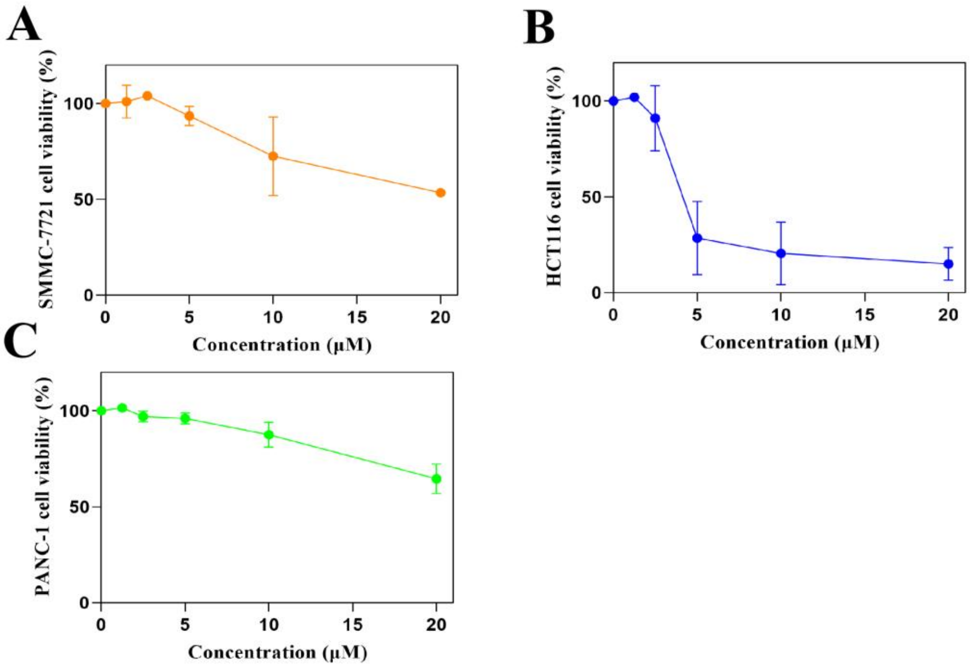

A
SMMC-7721 cell viability (%)
100
50
0
0
5
10
15
20
Concentration (μM)
B
HCT116 cell viability (%)
100
50
0
0
5
10
15
20
Concentration (μM)
C
PANC-1 cell viability (%)
100
50
0
0
5
10
15
20
Concentration (μM)

## Figure S6.

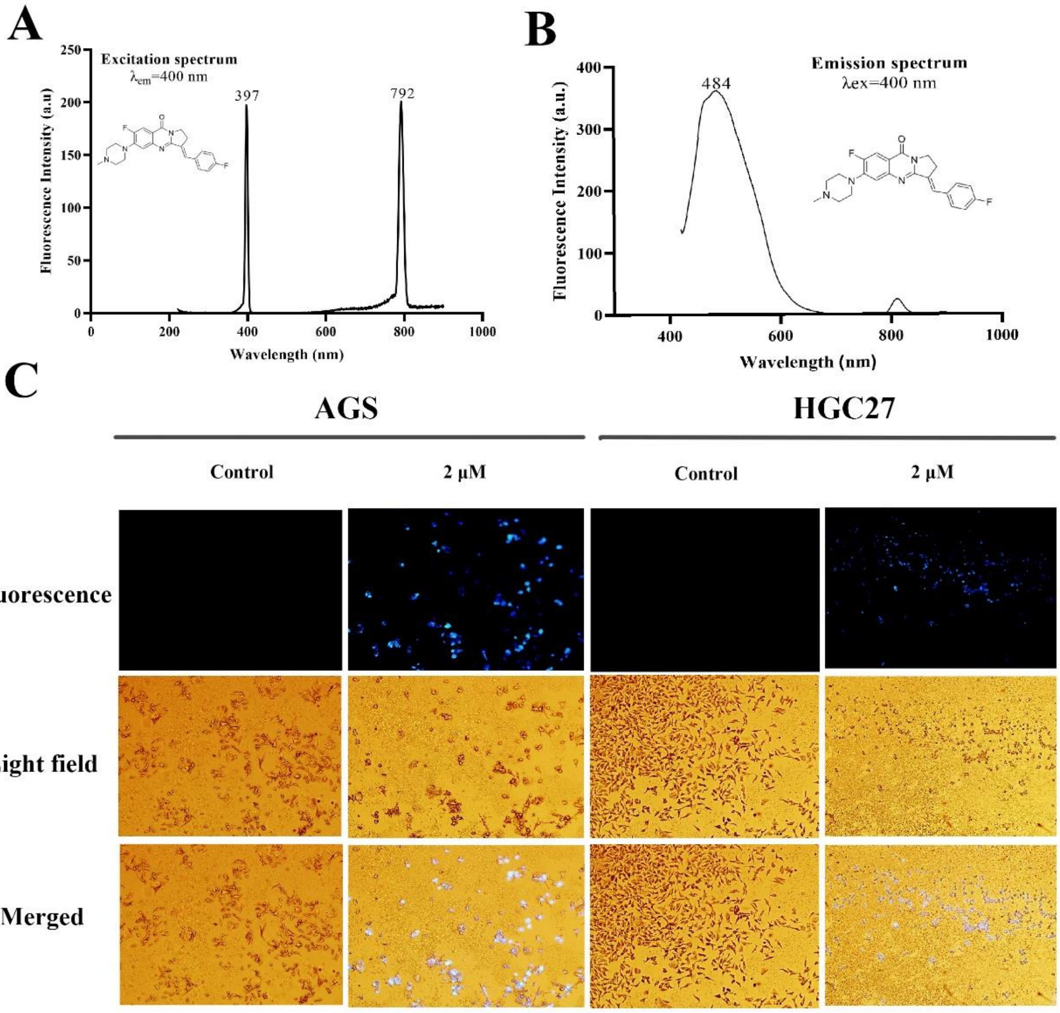
A
Excitation spectrum
$\lambda_{em}$=400 nm
397
792
Fluorescence Intensity (a.u)
Wavelength (nm)
B
Emission spectrum
λex=400 nm
484
Fluorescence Intensity (a.u.)
Wavelength (nm)
C
AGS
HGC27
Control
2 μM
Control
2 μM
Fluorescence
Light field
Merged
